# Discharge Planning: Screening Older Patients for Multidisciplinary Team Referral

**DOI:** 10.5334/ijic.2252

**Published:** 2016-10-10

**Authors:** Carolyn Hegarty, Clare Buckley, Rachel Forrest, Bob Marshall

**Affiliations:** 1 Hawke’s Bay District Health Board, Omahu Road, Hastings, New Zealand; 2 Eastern Institute of Technology, Private Bag 1201, Napier, New Zealand

**Keywords:** Elders Risk Assessment Index, Older adults/people, Emergency Department, Discharge planning, Multi-disciplinary team

## Abstract

The objective was to determine whether the Elders Risk Assessment Index can predict multi-disciplinary team referral of older patients (≥ 65 years) in Emergency Department same-day discharges.

The study identified 1,376 qualifying individuals from a regional New Zealand hospital database. Of these, 12.7 % were referred to the multi-disciplinary team. Univariate and multivariate analyses were used to explore associations between the Index, its components, and other demographic factors with referral. With every unit increase in the Index there was a 9% increase in the odds of being referred. When the components of the Index were analysed separately, an increased likelihood of being referred was associated with not being married, having had a previous hospital admission of more than five days, having chronic obstructive pulmonary disease, and being older. Conversely, a decreased likelihood was associated with having diabetes. When non-Index items were analysed it was found that females were more likely to be referred than males and that Māori were less likely to be referred than New Zealand Europeans.

With adaptation, the Elders Risk Assessment Index may provide a simple, cost-effective, and timely tool to assist in determining the need for multi-disciplinary team referral for older people who present to the Emergency Department.

## Keypoints

This research confirmed a significant association between the Elders Risk Assessment Index, its components and referral of older patients to the multi-disciplinary team.

The research supports the notion that with adaptation, the Elders Risk Assessment Index may provide a simple, cost-effective, and timely tool to assist in determining the need for multi-disciplinary team referral for older people who present to the Emergency Department.

The low referral rate (12.7%) suggests that there may be missed referral opportunities and this requires further research.

Research is needed to assess the variables of gender and ethnicity, and the importance of other factors such as mobility as predictors of multi-disciplinary team referral.

## Introduction

Effective discharge planning from the Emergency Department to home aids integration of care for older adults by facilitating appropriate use of social and support services, and any medical care that may be required [1]. Essential to integrating care in these social and support services is initial referral to a multi-disciplinary team whose role is to focus on safe, early discharge to home and to identify and overcome barriers that may impede this safe discharge [2]. The multi-disciplinary team assesses the patient’s needs, and identifies and integrates appropriate services to prevent both avoidable hospital admissions, and unsuccessful hospital discharges. While identifying individual needs and developing a discharge plan does not guarantee integrated care, without this degree of planning integrated care is unlikely [3,4]. Research has linked multi-disciplinary team referral and intervention in the Emergency Department with a reduction in hospital admission rates, and Emergency Department re-presentation [5,6,7,8]. Unfortunately, time and fiscal constraints often render it impractical for referral of every older adult who presents to the Emergency Department for multi-disciplinary team assessment [9,10]. Thus, the identification of vulnerable or at-risk older adults who are more likely to require multi-disciplinary team referral is essential at the point of entry to the Emergency Department system [4,11]. Delays in referral will compromise the ability of clinicians to be able to discharge older adults home within mandated time frames, and may also impact on the safe discharge of these patients and also result in avoidable admissions to hospital [10]. A literature review identified a number of existing guidelines for nurses and physicians based on either patient and/or caregiver reporting, or nurse reporting. Each guideline allows clinicians to identify need for referral based on their clinical judgement alone. Whilst these guidelines achieve the aim of identifying older-at-risk patients, they tend to be time-consuming to complete and it is not clear if the decision to refer is made because of the time-consuming reporting, or simply because of individual clinicians’ clinical judgement and decision-making.

Currently there are no Emergency Department-specific screening tools to determine the referral of older patients to the multi-disciplinary team and the literature suggests that the decision to refer is made in an ad hoc manner with referral frequently taking place after suggestion from a physician [12]. Screening tools for utilisation in the Emergency Department would need to be short and easy to administer at triage due to the busy nature of the department. An ideal screening tool would be able to identify those patients who are safe for discharge, those patients safe for discharge who will benefit from multi-disciplinary team assessment, and those not safe for discharge. Whilst there are a number of documented risk-assessment screening tools detailed in the literature [9,11,13,14,15], only two tools which screen administrative data available at triage were identified. These were the Silver Code tool [16] and the Elders Risk Assessment Index [17]. The former has been used to predict Emergency Department readmissions and future hospitalizations in patients discharged directly from the Emergency Department [16], however, it was not appropriate for this study as sufficient data to complete the Silver Code tool was not available in the administrative database of the regional New Zealand hospital.

The Elders Risk Assessment Index was developed to predict older patients at risk of re-hospitalisation and Emergency Department visits by calculating a risk score based on age, marital status, history of hospital admission, length of hospital stay in the two years prior to the current Emergency Department visit, and co-morbidities [17]. It has been validated as a predictor for hip fractures [18], and to predict mortality and Aged Care placement in community-dwelling adults [19]. An Elders Risk Assessment Index of 16 or more has also been hypothesised in the literature as a measuring instrument to select patients for referral to a transitional care programme for community dwelling adults [20]. However, the use of the Elders Risk Assessment Index to predict the need for multi-disciplinary team referral in the Emergency Department setting has not been documented.

The regional New Zealand hospital used in this study did not have formalised guidelines in the Emergency Department for the referral of older patients to the multi-disciplinary team (which consisted of a social worker, a physiotherapist, an occupational therapist, and a gerontology clinical nurse specialist) to assist clinicians in the referral decision-making process. This research aimed to ascertain if the Elders Risk Assessment Index could predict multi-disciplinary team referrals for same-day discharges from the Emergency Department to home for older people (≥65 years of age) for an 11-month period between 1 July 2011 and 31 May 2012.

## Method

The research was a retrospective, quantitative, observational study in which the Elders Risk Assessment Index for same-day discharge Emergency Department patients ≥65 years of age was calculated and associations between multi-disciplinary team referral and Elders Risk Assessment Index were explored.

### Study setting and population

The study sample was retrieved from a regional New Zealand hospital Emergency Department administrative database.

### Inclusion and exclusion criteria

The inclusion criteria were all presentations from patients ≥65 years of age who resided in the research District Health Board area who presented to the Emergency Department and were treated and discharged home on the same day during the specified period. Patients could have more than one presentation to the Emergency Department during the data collection period.

Palliative care patients, dialysis patients, residents of aged care facilities, and patients residing ≥100 km from the District Health Board Emergency Department were excluded. This last exclusion criterion represents the population who were unlikely to be discharged home due to geographical location.

### Ethical considerations

Ethical approval was obtained from the authors’ tertiary institution’s Research Ethics and Approval Committee as well as from the hospital research approval panel.

### Sources, collection, and management of data

All data for this study were abstracted from the District Health Board administrative database. The variables selected for collection within this study were based on the Elders Risk Assessment Index criteria (Table 1). Additional variables distinct from the Elders Risk Assessment Index were collected for each presentation to provide a demographic description of the sample including gender and ethnicity. Presentation date and time, and referral to the multi-disciplinary team (independent variable) were also abstracted from the database. All data were anonymised.

**Table 1:** Profile of the older (≥ 65 years old) patients presented to a regional NZ hospital Emergency Department (N=1376) showing percentage referred to the multidisciplinary team (MDT) within each group and the percentage of total referrals (n=175) along with the odds ratios of being referred for each group within each category.

Category^1^	Group	% (n)	% of total	% referred	Univariate Analyses^3^	
			Referrals (n)	to MDT^2^	P value	Odds ratio (95% CI)

Gender	Female	53.1 (730)	64.6 (113)	15.5	**0.001**	1.257 (1.112, 1.421)
	Male	46.9 (646)	35.4 (62)	9.6		0.729 (0.592, 0.897)
All European	Yes	89.4 (1230)	95.4 (167)	13.6	**0.005**	1.078 (1.038, 1.120)
	No	10.6 (146)	4.6 (8)	5.5		0.398 (0.199, 0.797)
Māori	Yes	8.5 (116)	3.4 (6)	5.2	**0.011**	0.374 (0.152, 0.814)
	No	91.6 (1260)	96.6 (169)	13.4		1.063 (1.028, 1.099)
Pacifica	Yes	1.2 (17)	0.0 (0)	0.0	0.113	.
	No	98.6 (1184)	100.0 (175)	12.9		1.014 (1.008, 1.021)
Other/missing	Yes	0.9 (13)	1.1 (2)	15.4	0.772	1.248 (0.279, 5.583)
	No	99.1 (1363)	98.9 (173)	12.7		0.998 (0.981, 1.015)

Married/De-facto	Yes	53.3 (733)	33.7 (59)	8.0	**<0.001**	0.601 (0.485, 0.744)
	No	46.7 (643)	66.3 (116)	18.0		1.511 (1.335, 1.709)
65–69 years	Yes	24.2 (333)	10.3 (18)	5.4	**<0.001**	0.392 (0.251, 0.614)
	No	75.8 (1043)	89.7 (157)	15.1		1.216 (1.145, 1.292)
70–79 years	Yes	42.4 (583)	33.7 (59)	10.1	**0.013**	0.773 (0.622, 0.960)
	No	57.6 (793)	66.3 (116)	14.6		1.176 (1.046, 1.322)
80–89 years	Yes	29.4 (405)	46.9 (82)	20.2	**<0.001**	1.742 (1.451, 2.093)
	No	70.6 (971)	53.1 (93)	9.6		0.727 (0.630, 0.839)
90 + years	Yes	4.0 (55)	9.1 (16)	29.1	**<0.001**	2.816 (1.608, 4.928)
	No	96.0 (1321)	90.9 (159)	12.0		0.936 (0.895, 0.985)
Previous LOS< 6 days	Yes	31.7 (436)	30.3 (53)	12.2	0.670	0.950 (0.747, 1.207)
	No	68.3 (940)	69.7 (122)	13.0		1.024 (0.921, 1.137)
Previous LOS> 5 days	Yes	22.2 (305)	37.7 (66)	21.6	**<0.001**	1.895 (1.518, 2.365)
	No	77.8 (1071)	62.3 (109)	10.2		0.778 (0.691, 0.876)
COPD	Yes	14.6 (201)	21.7 (38)	18.9	**0.004**	1.600 (1.167, 2.193)
	No	85.4 (1175)	78.3 (137)	11.7		0.906 (0.835, 0.982)
Cardiac conditions	Yes	39.2 (540)	43.4 (76)	14.1	0.225	1.124 (0.936, 1.351)
	No	60.8 (836)	56.6 (99)	11.8		0.922 (0.804, 1.058)
Stroke	Yes	10.1 (139)	13.1 (23)	16.5	0.153	1.361 (0.896, 2.068)
	No	89.9 (1237)	86.9 (152)	12.3		0.961 (0.905, 1.021)
Dementia	Yes	3.1 (43)	4.6 (8)	18.6	0.239	1.569 (0.740, 3.326)
	No	96.9 (1333)	95.4 (167)	12.5		0.983 (0.950, 1.017)
Cancer	Yes	17.2 (236)	17.1 (30)	12.7	0.998	0.999 (0.705, 1.416)
	No	82.8 (1140)	82.9 (145)	12.7		1.000 (0.931, 1.075)
Diabetes	Yes	19.0 (261)	14.3 (25)	9.6	0.091	0.727 (0.497, 1.064)
	No	81.0 (1115)	85.7 (150)	13.5		1.067 (0.998, 1.140)

^1^Gender and Ethnicity are not part of the Elders Risk Assessment Criteria. LOS = length of stay, COPD = Chronic Obstructive Pulmonary/Respiratory Disease. ^2^A P value <0.05 in the univariate analysis (Pearson’s Chi-square test or Fisher’s Exact Tests) indicates the percentage referrals to MDT for each group within the category is significantly different. ^3^2X2 contingency tables of MDT referral (yes, no) and category (yes, no) were analyzed using a Pearson’s Chi-square test and used to calculate the odds ratios.

The Elders Risk Assessment indices were calculated for each individual presentation during the study period. Each component of the index is associated with a value and these are summed together to determine the total Index value. The Index components and their associated values are as follows: Married, -1; Age 65–69 years, 0; Age 70–79 years, 1; Age 80–89 years, 3; Age 90+ years, 7; Admission to hospital 1–5 days in the previous 2 years, 5; Admission to hospital >5 days in the previous 2 years, 11; Diabetes, 2; Coronary artery disease/myocardial infarction/congestive heart failure, 3; Stroke, 2; Chronic obstructive pulmonary/respiratory disease, 5; Cancer -excluding non-melanomatous skin cancer, 1; Dementia, 3.

The original Elders Risk Assessment age-range criterion commenced at age 70. However, similar to Boyd et al. [9], a modification was made to the age-range of the original Elders Risk Assessment scoring system to extend the age-range to include people aged ≥65 years. This extension of age was in response to research which identified ethnic inequalities in relation to increased mortality; and also a higher incidence of cancer and cardiovascular disease in Māori and Pasifika people in the ≥65 years age range [21].

### Data analysis

For each presentation to the Emergency Department, age group, marital status, length of stay, and the presence of various diseases were used to calculate the Elder Risk Assessment Index. Two additional non-Index factors, gender and ethnicity, were also explored as potential predictors of older patients’ referral to the multi-disciplinary team as this information is routinely collected and readily available in the administrative database. The data were analysed using the Statistical Package for Social Sciences™ version 22. Descriptive statistics were used to examine the demographic characteristics of the presentation data set, with student t-Test and Pearson’s correlation being used to determine if an association between Elders Risk Assessment Index and multi-disciplinary team referral existed. Both univariate (Pearson’s Chi-square), and multivariate (binary logistic regression) analyses together with their odds ratios were then used to explore the nature of the associations between the Elders Risk Assessment Index, its components along with gender and ethnicity with multi-disciplinary team referrals. Several multivariate models were analysed. Those Index components in the univariate analyses (Pearson Chi-square) that had a P value of less than 0.100 were included in forward and backward stepwise binary logistic regressions. These analyses were repeated including the non-Index factors that also had a P value less than 0.001.

In order to ascertain whether an Elders Risk Assessment Index of 16 was an appropriate threshold to select patients for referral to the multi-disciplinary team, a 2×2 contingency table of multi-disciplinary team referral (yes, no) and Index group (<16, ≥16) was analysed using a Pearson’s Chi square test and calculation of the odds ratio.

## Results

**Figure 1:**
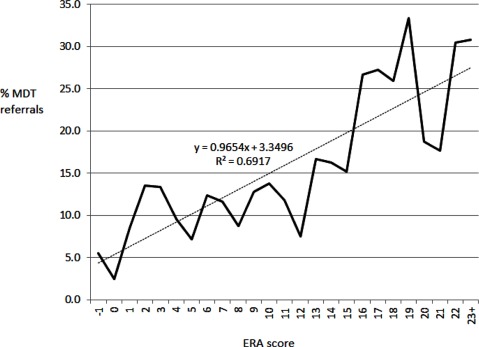
The percentage of multi-disciplinary team referrals recorded for each Elders Risk Assessment Index (solid line) with the linear trendline (dotted).

The data set comprised 1,376 presentations, predominantly females (53.1%), individuals between the ages of 70–79 years (42.4%), and living with a partner (53.3%) (Table 1). The three most prevalent disease types were cardiac conditions (coronary artery disease, myocardial infarction, and/or congestive heart failure) at 39%, diabetes (19%) and cancer (17%) (Table 1). Only 12.7% of the participants were referred to the multidisciplinary team. As expected, the mean Elders Risk Assessment Index for those referred to the multi-disciplinary team was significantly higher than that of those who were not referred (Student T-test, p < 0.001; referred 10.95 ± 0.181, not referred 7.38 ± 0.520, n=1376). Figure 1 shows the positive correlation between the Index score and percentage of multi-disciplinary team referrals (P<0.001, r=0.831) can be seen.

Gender is not part of the Elders Risk Assessment Index criteria. However, in this study gender was found to be associated with multi-disciplinary team referral with 64.6% of all the referred patients being female (Pearson’s Chi-square, P<0.001). Ethnicity data were also collected and Māori only comprised 3.4% of the multi-disciplinary team referrals, which is below the 5.8% of Māori in the region [22]. Certain ethnicities (European and Maori) were found to be associated with referral in the univariate analyses (Table 1).

The percentage referred to the multidisciplinary team for each of the Elders Risk Index components and those non-Index factors being investigated are shown in Table 1, along with the odds ratios for being referred for each group within each component category. Significantly different odds ratios (determined by Pearson’s Chi-square) were observed for gender, various ethnicities (European and Maori), marital status, each of the age group, previous hospital admission of more than five days, and the co-morbidities chronic obstructive pulmonary disease, with diabetes tending towards significance (Table 1).

A binary logistic regression of multi-disciplinary team referral and the Elders Risk Assessment Index confirmed a significant association between the two variables and revealed that with every one unit increase in the Index there is an 8.5% increase in the odds of being referred (Table 2). When gender (male, female) and ethnicity group (entered as one variable) were added to the binary logistic regression model with the Index and a stepwise approached used, both were retained along with the Index, with females being almost twice as likely to referred and Māori being more than 30% less likely to be referred than Europeans (Table 2).

**Table 2:** Odds ratios of a multidisciplinary team (MDT) referral and their significance (P value) as determined by a forward and backward stepwise binary logistic regressions for those Elders Risk Assessment (ERA) Criteria in Table 1 that had a P<0.100 in the univariate analyses.

Factors included in the Model	P value	Odds Ratio	95 % Confidence Interval	
			Lower	Upper

*Model: Index only*				
Index	<0.001	1.085	1.060	1.112

*Model: Index with gender and ethnicity*				
Index	<0.001	1.091	1.065	1.118
Gender (female/male)	0.001	1.805	1.287	2.532
Ethnicity (reference: European)	0.026			
Maori	0.002	0.269	0.115	0.628
Pacifica	0.998	0.000	0.000	–
Other	0.862	1.149	0.237	5.571

*Model: Index components from univariate analyses with P*<*0.1*				
Married	0.001	0.538	0.378	0.766
LOS>5days	<0.001	2.104	1.476	3.001
COPD	0.020	1.638	1.080	2.484
Diabetes	0.027	0.589	0.369	0.941
Age groups (reference: 65–70)	<0.001			
90+	<0.001	4.662	2.132	10.192
80 – 89	<0.001	3.299	1.902	5.722
70 – 79	0.051	1.736	0.997	3.024

*Model: Index components from univariate analyses with P*<*0.1 with gender and ethnicity*				
Married	0.002	0.554	0.384	0.798
LOS>5days	<0.001	2.162	1.512	3.092
COPD	0.005	1.842	1.203	2.821
Diabetes	0.047	0.618	0.385	0.993
Age groups (reference: 65–70)	<0.001			
90+	0.001	3.936	1.789	8.660
80 – 89	<0.001	2.835	1.624	4.950
70 – 79	0.084	1.635	0.935	2.859
Gender (female/male)	0.024	1.505	1.056	2.143
Ethnicity (reference: European)	0.103			
Maori	0.014	0.337	0.141	0.805
Pacifica	0.998	0.000	0.000	–
Other	0.688	1.388	0.281	6.861

When the components of the Elders Risk Assessment Index that had a P<0.100 in the univariate analyses (Table 1) were included in a multivariate analysis (forward and backward stepwise binary logistic regression); marital status, age groups (included as one variable), previous hospital admission of longer than five days, and the comorbidities chronic obstructive pulmonary/respiratory disease and diabetes were retained indicating they were each independently associated with multi-disciplinary team referral (Table 2). When gender (male, female) and ethnicity group (entered as one variable) were also included in the models both were retained as expected given the univariate analyses results (Table 2). Collectively the univariate and multivariate analyses indicate that increasing age, a previous hospital visit of greater than five days, having chronic obstructive pulmonary/respiratory disease, being female and being European are the strongest predictors of referral; whereas, being Maori, married and having diabetes are the strongest predictors of non-referral.

An Index of 16 has been suggested as a threshold to select patients for referral to a transition care programme for community dwelling adults and therefore could act as a potential threshold for multi-disciplinary team referral in the Emergency Department. A significant difference in the proportion of multi-disciplinary team referrals was observed between the two Index groups, with 27.1% (57 out of 210) of those with an Index of 16 or more being referred to the multi-disciplinary team, compared to 10.1% (118 out of 1166) of those with an Index of less than 16 (Pearson’s Chi-square, P<0.0001). Those with an Index or 16 or more were 3.3 times more likely to be referred than those with an Index ≥16 (≥16, Odds ratio 2.56, 95% CI 1.972–3.311; <16, Odds Ratio 0.77, 95% CI 0.695–0.858). Of note is that 72.9% of those scoring 16 or more were not referred indicating there may be missed referral opportunities.

## Discussion

The findings demonstrate a significant association between multi-disciplinary team referral and the Elders Risk Assessment Index and more specifically with particular components of the Index. In the absence of a purposefully designed risk assessment tool, the Elders Risk Assessment Index could be utilised in the Emergency Department to aid in determining the referral of older patients to the multi-disciplinary team. Syed [20] suggested that patients with an Elders Risk Assessment Index of 16 or more should be referred to a transitional care programme for community dwelling adults. This study suggests that an Elders Risk Assessment Index of 16 may be an appropriate threshold for selecting patients for referral to the multi-disciplinary team, however, only 27% of those with an Index of 16 or more were referred, thus the number being referred would increase dramatically.

Whilst this study supports the notion of using of the Elders Risk Assessment Index as a screening tool to aid in determining the referral of older adults to the multi-disciplinary team it also indicates that a more accurate screening tool could be developed. Gender is not included in the variables for Elders Risk Assessment Index calculation because Crane et al. [17] found that gender was not statistically significant in predicting risk of Emergency Department encounters and hospitalisation of elderly patients. However, in this study, the results show that gender was significantly associated with multi-disciplinary team referral, with females being more likely to be referred than males. Our data suggest that including gender would improve the ability of the Elders Risk Assessment to predict need for multi-disciplinary team referral. However, further research would be needed to confirm this as it may be that females are over-represented in the referred group due to other extraneous variables, and including gender in the Elders Risk Assessment Index would merely serve to increase the gender bias.

Whilst the literature identifies mobility as a key indicator of multi-disciplinary team referral [23], the Elders Risk Assessment does not assess mobility. This may be a factor in why patients who had a lower Elders Risk Assessment Index in this study were referred to the multi-disciplinary team. A modification to the Elders Risk Assessment Index to include a history of falls may increase the applicability in the Emergency Department setting. However, it is acknowledged that falls are consistently underrepresented in electronic medical records with mobility not always being assessed by providers; let alone recorded in electronic records [24,25]. In light of this, a modification to the Elders Risk Assessment Index to include mobility or history of falls is unlikely to contribute to the applicability of the Elders Risk Assessment Index as a predictor of multi-disciplinary team referral.

Within the New Zealand setting, inequalities have been associated with increased mortality and morbidity in Māori and Pacifika people compared with Europeans [21,26]. This study found ethnic inequalities may also be present in relation to patient selection for multi-disciplinary team referral as no Pacifika patients were referred and Māori represented just 3.4% of all multi-disciplinary team referrals, which is below the 5.8% representation of Māori aged 65 years and older in the Hawke’s Bay region’s population [22]. Thus, any modification of the Index for use in New Zealand should review whether inclusion of ethnicity is appropriate.

Finally, this study also found that 73% of the patients who scored ≥16 were not referred to the multi-disciplinary team. This may indicate that these patients were missed referrals and as such support the suggestion that there may be inconsistencies in patient selection for multi-disciplinary team referral. This would appear to support the need for a more formalised referral system over and above the current ad hoc system.

## Conclusion

In the absence of a purpose-specific tool, this study suggests that the Elders Risk Assessment Index could be used to aid in the decision to refer an older adult to the multi-disciplinary team. However, as nearly three quarters of older adults who scored 16 or more on the Elders Risk Assessment Index were not referred to the multi-disciplinary team, it is clear that more research is needed to establish the referral score that would allow the Elders Risk Assessment Index to be used with confidence. We do not recommend that the Elders Risk Assessment Index be used exclusively, however, this research suggests that the Elders Risk Assessment Index is a partial predictor of multi-disciplinary team referral and that there is scope to create a New Zealand-based tool for use in the Emergency Department. Further research is also needed to assess the variables of gender and ethnicity, and the import of mobility as predictors of multi-disciplinary team referral. This research provides valuable information that can inform future research to develop a tool to aid in decision-making around the referral of older adults for multi-disciplinary team review. As noted initially, while identifying individual needs and developing a discharge plan does not guarantee integrated care, without this degree of planning integrated care is unlikely.

## Supplementary Files

The supplementary files for this article can be found as follows:

- **Supplementary File 1: Appendix 1. **ijic-16-4-2252-s1.pdf
