# Supplementary material for: Discharge Planning: Screening Older Patients for Multidisciplinary Team Referral
Source: Int J Integr Care. 2016 Oct 10;16(4):1. doi: 10.5334/ijic.2252 (PMC5388067; doi:10.5334/ijic.2252)
Supplement: Supplementary file 1 [file ijic-16-4-2252-s1.pdf]

Appendix 1: Elders Risk Assessment Index Criteria and Score

Criteria	Score
Married	-1
Age 65-69	0
Age 70-79	1
Age 80-89	3
Age 90+	7
Admission to hospital 1-5 days previous 2 years	5
Admission to hospital >5 days previous 2 years	11
Diabetes	2
Coronary Artery Disease, Myocardial Infarction, Congestive Heart Failure	3
Stroke	2
Chronic Obstructive Respiratory Disease	5
Cancer (excluding non-melanomatous skin cancer)	1
Dementia	3
